# Hepcidin-Dependent Regulation of Erythropoiesis during Anemia in a Teleost Fish, *Dicentrarchus labrax*

**DOI:** 10.1371/journal.pone.0153940

**Published:** 2016-04-21

**Authors:** João V. Neves, Carolina Caldas, Miguel F. Ramos, Pedro N. S. Rodrigues

**Affiliations:** 1 Instituto de Investigação e Inovação em Saúde, Universidade do Porto, Porto, Portugal; 2 Iron and Innate Immunity, Instituto de Biologia Molecular e Celular (IBMC), Universidade do Porto, Porto, Portugal; 3 Instituto de Ciências Biomédicas Abel Salazar (ICBAS), Universidade do Porto, Porto, Portugal; National Cheng Kung University, TAIWAN

## Abstract

Anemia is a common disorder, characterized by abnormally low levels of red blood cells or hemoglobin. The mechanisms of anemia development and response have been thoroughly studied in mammals, but little is known in other vertebrates, particularly teleost fish. In this study, different degrees of anemia were induced in healthy European sea bass specimens (*Dicentrarchus labrax*) and at pre-determined time points hematological parameters, liver iron content and the expression of genes involved in iron homeostasis and hematopoiesis, with particular attention on hepcidins, were evaluated. The experimental anemia prompted a decrease in *hamp1* expression in all tested organs, in accordance to an increased need for iron absorption and mobilization, with slight increases in *hamp2* in the kidney and intestine. The liver was clearly the major organ involved in iron homeostasis, decreasing its iron content and showing a gene expression profile consistent with an increased iron release and mobilization. Although both the spleen and head kidney are involved in erythropoiesis, the spleen was found to assume a more preponderant role in the recovery of erythrocyte levels. The intestine was also involved in the response to anemia, through the increase of iron transporting genes. Administration of Hamp1 or Hamp2 mature peptides showed that only Hamp1 affects hematological parameters and liver iron content. In conclusion, the molecular mechanisms of response to anemia present in sea bass are similar to the ones described for mammals, with these results indicating that the two hepcidin types from teleosts assume different roles during anemia.

## Introduction

Anemia is one of the most common disorders of the blood, resulting from an abnormally low level of red blood cells or hemoglobin. Anemia symptoms can range from asymptomatic to weakness, shortness of breath, fatigue, and in the most severe cases, organ damage and heart failure, leading to death. Although numerous types of anemia have been characterized, they can be divided into three major groups: caused by blood loss, by excessive hemolysis or by impaired erythropoiesis. There are several causes that can lead to the development of anemia, but the most common is iron deficiency [1]. Iron is essential for the synthesis of hemoglobin, the key component of red blood cells responsible for oxygen binding and transport [2, 3], as well as numerous other cellular processes [4–8]. Iron deficiency is usually derived from insufficient iron uptake (low dietary iron), deficiencies in iron absorption, storage or transport, or significant blood loss. When anemia is established, one of the results is a decrease in the levels of oxygen that the blood is able to carry, which can eventually lead to hypoxia. In hypoxia, less oxygen is available for normal cellular processes, leading to decreased energy production, compromised cell proliferation and repair, reduced muscular activity and, in extreme cases, severe oxygen deprivation to the brain, which could lead to death.

As such, it is clear that there is a tight interconnection between red blood cell levels, oxygen homeostasis and iron metabolism. The existing studies addressing the mechanisms involved in the establishment of anemia and the genes involved in hematopoiesis are mostly focused on mammals and there are still numerous gaps in the understanding of these processes in lower vertebrates, particularly fish. With the continuous depletion of the natural fish stocks, many believe that in the years to come aquaculture will represent one of the major food sources. Consequently, a better understanding of the mechanisms of iron metabolism in response to anemia and to the need for an enhanced erythropoiesis is crucial for the increased welfare of aquaculture species.

Assuming a central role in iron metabolism is hepcidin, a small cysteine-rich peptide that is considered to be the key regulator of iron metabolism [9–12]. Hepcidin is mostly produced in the liver hepatocytes, but it has also been described in other cell types and tissues. As an iron metabolism regulator, hepcidin synthesis is regulated by several stimuli through a myriad of pathways (reviewed in [13, 14]), being induced by elevated iron levels and infection/inflammation and decreased by low iron levels, anemia and hypoxia. When iron stores are adequate or high, or during infection, hepcidin binds to the iron exporter ferroportin, causing its internalization and degradation, thus blocking the release of iron from macrophages, hepatocytes and enterocytes, and in the later, also leading to decreased iron absorption [15, 16]. Inflammation also leads to a limitation in iron availability not only for pathogens but also for normal erythropoiesis, which could lead to the so called anemia of inflammation [17, 18]. On the other hand, when iron or oxygen levels are low, hepcidin production is attenuated or suppressed, increasing cellular iron efflux and consequently intestinal uptake.

An interesting fact about fish hepcidins is that, contrary to mammals, where a single gene exists (with the mouse being the sole known exception [19]) and performs both as an antimicrobial peptide and iron regulator, many teleost fish present two types of hepcidin (commonly referred to as hamp1 and hamp2) [20–24] and although the full extent of their roles remains unclear, one is usually more associated with iron metabolism regulation and the other with the antimicrobial response.

With the present work, we intend to clarify the molecular mechanisms of response to anemia in a commercially relevant teleost fish, the European sea bass (*Dicentrarchus labrax*), by investigating the expression of several genes known to be involved in iron homeostasis and hematopoiesis, with a particular focus in understanding the roles of the different hepcidin genes.

## Materials and Methods

### Animals

European sea bass (*Dicentrarchus labrax*), with an average weight of 50g, were provided by a commercial fish farm in the south of Portugal (Piscicultura do Vale da Lama, Lagos, Portugal). Fish were kept in 500 liters recirculating sea water tanks at 21±1°C, with a 12-hour light/dark cycle and fed daily to satiation. Before each treatment, fish were anaesthetized with ethylene glycol monophenyl ether (2-phenoxyethanol, 0.3 ml/liter, Merck, Algés, Portugal). All animal experiments were carried out in strict compliance with national and international animal use ethics guidelines, approved by the animal welfare and ethic committee of the Instituto de Biologia Molecular e Celular (IBMC), with permit ref. Ofício Circular n° 99, 0420/000/000 of 09/11/2009 from the Direcção Geral de Alimentação e Veterinária (DGAV), Portuguese Ministry of Agriculture and Sea, and conducted by FELASA Category C/DGAV certified investigators.

### Experimental anemias

Fish were individually weighted and bled from the caudal vessels the equivalent v/w of either 1% (“light” anemia) or 2.5% (“severe” anemia) body mass. Control fish were subjected to the same manipulation (anesthesia, weighting, pinching) but no blood was removed. One, four, seven and fourteen days after treatment, four fish from each of the experimental groups were anaesthetized and blood was drawn from the caudal vessels for evaluation of hematological parameters. Subsequently, fish were euthanized with an overdose of anesthetic, dissected and major tissues involved in iron homeostasis and hematopoiesis (liver, spleen, head kidney and intestine) were collected, snap frozen in liquid nitrogen and stored at -80°C until further use.

### Hematological parameters and liver iron content

To determine the impact of the experimental bleeding on the hematological parameters, peripheral blood was drawn from the caudal vessels at each experimental time point in terminally sampled animals. For red blood cell counts and hematocrit determination, 150 μl samples of blood were used in 1:1 dilutions with heparin in PBS (1000 units/ml). For determination of serum parameters, non-heparinized blood was transferred into 1.5 ml microcentrifuge tubes, and allowed to clot for 8 h at 4°C. The samples were centrifuged twice at 16000×g until a clear serum was obtained. Serum iron and transferrin saturation were determined by the Liquid Ferrozine® method (Thermo Electron, Victoria, Australia) according to the manufacturer’s specifications.

Non-heme iron was measured in livers by the bathophenanthroline method [25]. Briefly, liver samples with an average weight of 100 mg were placed in iron-free Teflon vessels (ACV-Advanced Composite Vessel, CEM Corporation, Matthews NC, USA) and dried in a microwave oven (MDS 2000, CEM Corporation). Subsequently, dry tissue weights were determined and samples digested in an acid mixture (30% hydrochloric acid and 10% trichloroacetic acid) for 20 h at 65°C. After digestion, a chromogen reagent (5 volumes of deionised water, 5 volumes of saturated sodium acetate and 1 volume of 0.1% bathophenanthroline sulfonate/1% thioglycollic acid) was added to the samples in order to react with iron and obtain a colored product that was measured spectrophotometrically at 535 nm. The *extinction coefficient* for bathophenanthroline is 22.14 mM^-1^cm^-1^.

### RNA isolation and cDNA synthesis

Total RNA was isolated from liver, spleen, head kidney and posterior intestine with the PureLink RNA Mini Kit protocol for animal tissues (Invitrogen, Life Technologies) with the optional on-column PureLink DNase treatment (Invitrogen), according to the manufacturer’s instructions. Total RNA quantification was performed using a NanoDrop 1000 spectrophotometer (Thermo Scientific, Waltham MA, USA) and quality was assessed by running the samples in an Experion Automated Electrophoresis Station (Bio-Rad). For all samples, 1.25 μg of each were converted to cDNA by Thermoscript™ and an oligo (dT) 20 primer (Invitrogen), for 40 min at 50°C, according to the manufacturer’s protocol.

### Analysis of gene expression by quantitative RT-PCR

Relative levels of several genes mRNAs were quantified in relevant organs of untreated, control and anemic fish, by real-time PCR. Genes analyzed include genes involved in iron homeostasis and regulation (*hamp1*, *hamp2*, *tmprss6*, *hjv*, *bmp6*, *bmpr2*, *smad4*, *smad1/5/8*, *tfr1* and *tfr2*), hematopoiesis (*epo*, *epor*, *hgb* and *gata2*), hypoxia (*hif1a*), iron uptake (*slc11a2a* and *slc11a2b*), iron storage (*fth*), iron export (*fpn*) and iron transport (*tf*). One μl of each cDNA sample was added to a reaction mix containing 10 μl iQ SYBR Green Supermix (Bio-Rad), 8.5 μl of _dd_H_2_0 and 250 nM of each primer (Table 1), making a total volume of 20 μl per reaction. A non-template control was included for each set of primers. The cycling profile was as follows: 94°C for 3.5 min, 40 cycles of 94°C for 30 s, 59°C for 30 s and 72°C for 30 s. Samples were prepared in duplicates, a melting curve was generated for every PCR product to confirm the specificity of the assays and a dilution series was prepared to check the efficiency of the reactions. β-actin was used as the housekeeping gene. The comparative CT method (2^-ΔΔCT^ method) based on cycle threshold (CT) values was used to analyze gene expression levels.

**Table 1:** Primers used for gene expression analysis.

	Forward (5'→3')	Reverse (5'→3')
*actb*	CAGAAGGACAGCTACGT	GTCATCTTCTCCCTGTTGGC
*hamp1*	CATTGCAGTTGCAGTGACACT	CAGCCCTTGTTGCCTCTG
*hamp2*	CTGCTGTCCCAGTCACTGA	ACCACATCCGCTCATATTAGG
*bmp6*	AAGCAGCCTTTCATGGTGGC	GGTTCATCAGGTGCACCAG
*bmpr2*	GCTGTAGCAGCCTTCTTTGG	CTCCTTTGAAGAGCTCGGTGT
*epo*	AGGCCAATCTGTGACCTGAG	GCAGTGCTGTGTTGGTGACT
*epor*	GCCTATGTCACCCTCAATGC	GAGTCTGCCACTGCCATGTA
*fpn*	GGCCTACTACAACCAGAACAT	AGGCCGCACTTCTTGCGAA
*fth*	AACCATGAGTTCTCAGGTGAG	TTAGCTGCTCTCTTTGCCCAG
*gata2*	CCCTGACCATGAAGAAGGAAG	TAGGCAGCATGTGTCCAGAG
*hgb*	CCAGGCTTTGACCAGACTTC	TGGACATCAGGGGTGAACTG
*hif1a*	AGCGGAGGAAGGAGAAGTC	CCATGAGAAAACCCTCCAGAG
*hjv*	AGGGCATCGAGGACCTGCT	CGCTCACCACCGAGCCAT
*slc11a2a*	CGCGTTCAACCTCCTCTCCTCT	AGCCCTCGCAGTACGGCACA
*slc11a2b*	TGCTCTCAACCTTCTCTCTGTG	AGCCGGCGCAGTAAGGTAAG
*smad4*	CAGTGCCACAGACAGATGCA	TGTGCAGGACCTCGTCCAG
*smad1/5/8*	CCATCGTCTACTACGAACTCAAC	GTGACGTCCTGTCGGTGATA
*tf*	CAACAGTATGGGTGCTGACG	ACTGGCAGAGCACTTGGACT
*tfr1*	CTCCTTCAACCACACCCAGT	GACCAGTACCGAGGTTCCAA
*tfr2*	GCCTACTTCAGTCTGGACCA	CCTCTGGACTGCAGCTCTG
*tmprss6*	CGCACTAATCTCCAGCCAGT	ATTCTGGGAGTGACCAGGTG

### Peptide administration

To evaluate the biological effects of sea bass hepcidin on the hematological parameters and liver iron content, synthetic peptides coding for the predicted mature peptides of Hamp1 (QSHLSLCRWCCNCCRGNKGCGFCCKF), Hamp2.1 (HSSPGGCRFCCNCCPNMSGCGVCCTF), and Hamp2.2 (HSSPGGCRFCCNCCPNMSGCGVCCRF) were commercially produced in the oxidized, folded form (with disulfide bonds) (Bachem AG, Bubendorf, Switzerland) [22] and administered to healthy sea bass. Briefly, peptides were diluted in 1× PBS to a final concentration of 100 μM and each fish was i.p. injected with 100 μl. Control animals received a similar volume of saline. At 1, 4, 7, 10 and 14 d post peptide administration, four fish from each group were anesthetized, blood was drawn from the caudal vessels for evaluation of hematological parameters and liver collected for liver iron determination, as previously described.

### Statistical analysis

Statistical analysis was carried out using GraphPad Prism 5 (GraphPad Software, Inc). Data normality was checked by performing Kolmogorof-Smirnoff test and Student’s T-test was used for estimating statistical significance. Multiple comparisons were performed with Two-way ANOVA and *post hoc* Tukey test. A p value of less than 0.05 was considered statistically significant.

## Results

### Hematological parameters and liver iron

**Fig 1:**
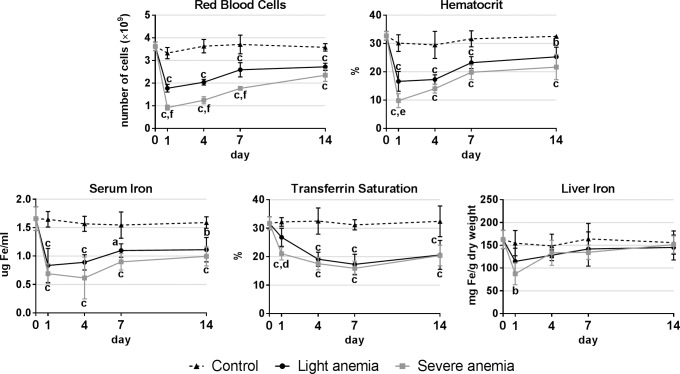
Hematological parameters and liver iron content. Results are presented as mean ± SD (n = 4). Differences were considered significant between control and treated groups as a, p<0.05; b, p<0.01; c, p<0.001; and between treated groups as d, p<0.05; e, p<0.01; f, p<0.001.

Hematological parameters and liver iron content were measured to validate and follow the progression of the experimental models of anemia (Fig 1). Similar significant decreases were observed in red blood cell numbers, hematocrit, serum iron and transferrin saturation in both light and severe anemia groups, although all parameters were consistently lower in the severe anemia group. Liver iron content was significantly reduced in the severe anemia group 1 day after anemia induction, whereas no significant variations were observed for the light anemia group.

### Gene constitutive expression

Constitutive expression of several genes was evaluated in relevant organs involved in iron homeostasis and hematopoiesis, including the liver, spleen, head kidney and posterior intestine. Overall, most genes presented the highest expression in the liver, with some exceptions (Table 2), with noticeable genes being *hamp1*, *hamp2*, *fth*, *tf*, *slc11a2b* and *tmprss6*. Also of relevance are the high levels of *epor*, *hgb* and *tfr1* in the head kidney, and of *slc11a2a* in the posterior intestine.

**Table 2:** Constitutive expression of several iron-related genes in sea bass liver, spleen, head kidney and posterior intestine, measured by real-time PCR. Each sample was normalized to beta actin, calculated by the comparative CT method (2^-ΔΔCT^). Values are presented as means (n = 4). N.D.–not detected.

	Liver	Spleen	Head Kidney	Posterior Intestine
*hamp1*	1,000,000	15,000	13,500	24,000
*hamp2*	200,000	20,000	2,500	12,000
*bmp6*	33,000	3,000	700	9000
*bmpr2*	25,000	900	900	1,400
*epo*	900	560	40	110
*epor*	8,100	22,000	46,000	1,700
*fpn*	8,800	8,500	3,800	9,200
*fth*	7,830,000	460,000	1,100,000	1,470,000
*gata2*	3,300	11,000	7,000	9,600
*hgb*	460,000	1,000,000	11,500,000	75,000
*hif1a*	160	50	40	70
*hjv*	19,300	1	N.D.	2
*slc11a2a*	2,200	260	290	10,000
*slc11a2b*	150,000	300	250	2,900
*smad4*	5,200	600	200	140
*smad5*	2,800	200	250	500
*tf*	9,000,000	10	1	6
*tfr1*	80,000	4,500	36,000	7,300
*tfr2*	6,500	90	50	260
*tmprss6*	900,000	400	200	30

### Gene expression under experimental conditions

#### *Hamp1* and *hamp2* expression in the liver, spleen, head kidney and posterior intestine

**Fig 2:**
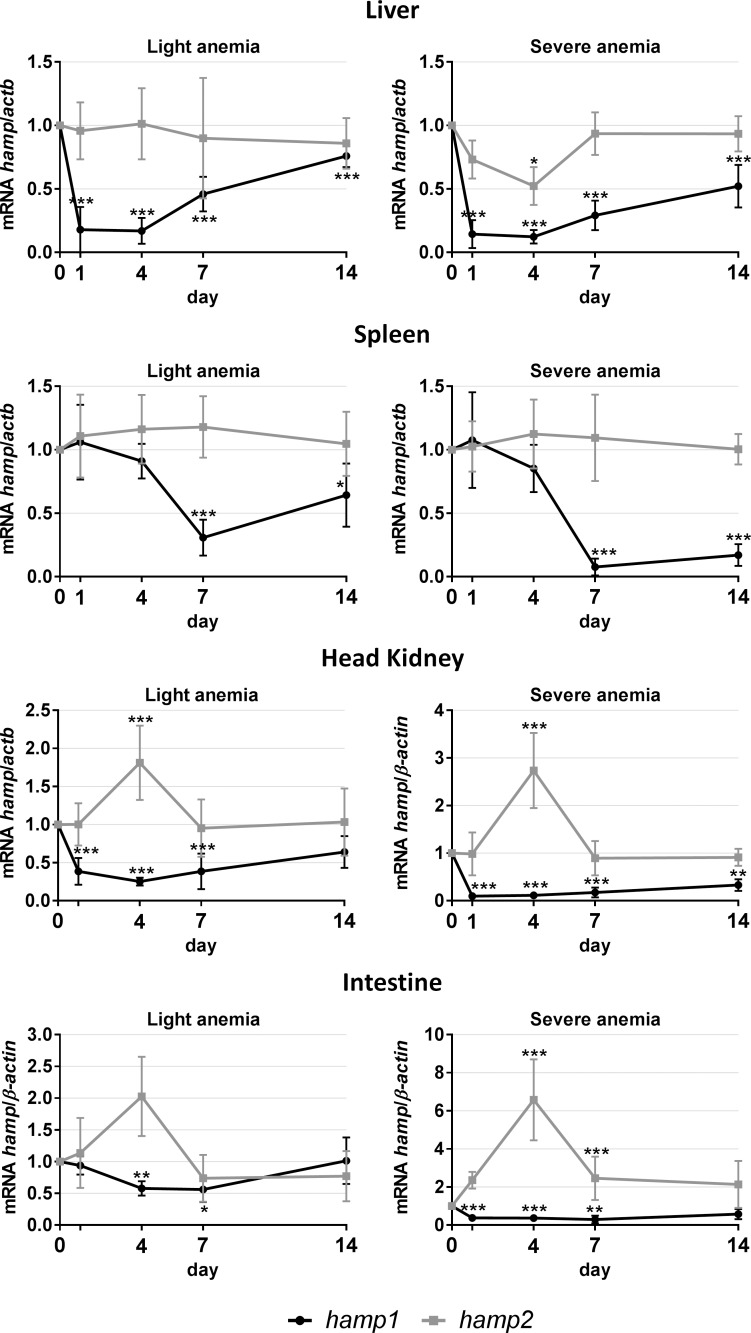
*Hamp1* and *hamp2* mRNA expression. Measured in in the liver, spleen, head kidney and posterior intestine by real-time PCR, after 1, 4, 7 and 14 of experimental anemias. Results are presented as mean ± SD (n = 4). Differences were considered significant between control and treated groups as a, p<0.05; b, p<0.01; c, p<0.001.

Similar patterns of expression were observed in the liver, spleen, head kidney and intestine for both experimental anemias (Fig 2). In the liver, *hamp1* expression was found to be significantly decreased throughout the whole experiment, with minimum levels reached as soon as day 1 and a gradual recovery towards day 14. In turn, *hamp2* was only significantly decreased at day 4 in the severe anemia group. In the spleen, no significant variations were observed in *hamp1* expression up to day 7, when an abrupt decrease occurred, followed by a slight recovery towards day 14. No changes were observed for *hamp2*. In the head kidney, *hamp1* expression dropped significantly at day 1 and gradually recovered up to day 14. *Hamp2* on the other hand, was increased at day 4 in the severe anemia group, returning to normal levels at day 7. Although not significant, the same tendency was observed for the light anemia group. In the intestine, a decrease in *hamp1* was observed as soon as day 1 way for the severe anemia group, and only at day 4 in the light anemia group, with both gradually recovering towards day 14. *Hamp2* expression was increased at day 4 in the severe anemia group, kept at still higher than normal levels at day 7 and returned to normal levels at day 14.

#### Gene expression in the liver

**Fig 3:**
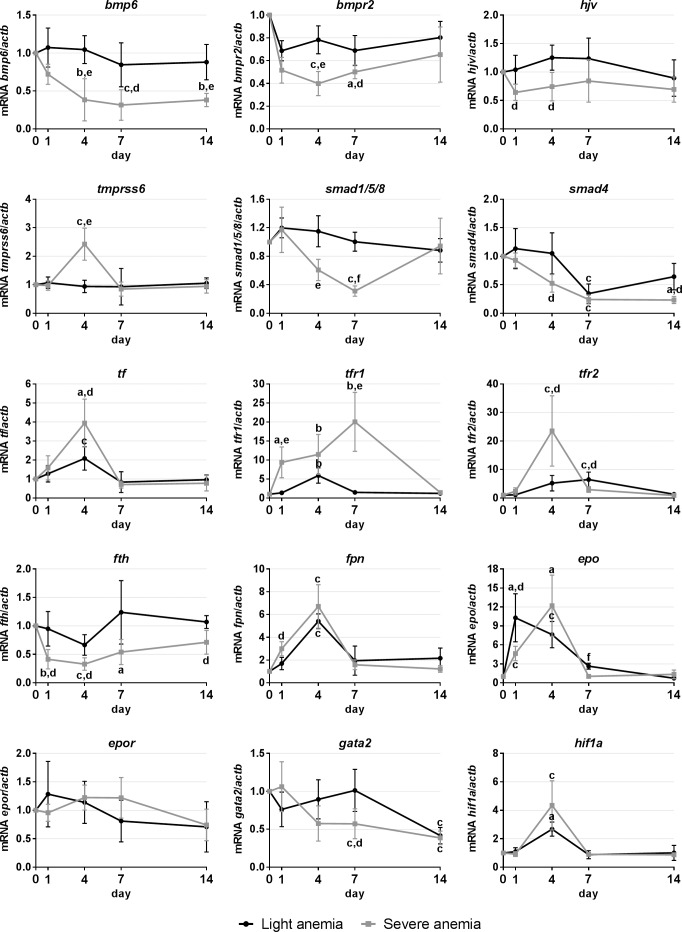
Gene expression in the liver, after 1, 4, 7 and 14 days of experimental anemias. Genes analyzed include genes encoding for hematopoietic transcription factors (*gata2*) and erythropoiesis-related factors (*epo/epor*), from the two major pathways of iron-sensing (*tmprss6*/*hjv*/*bmp6*/*bmpr2*/*smad* and *tf*/*tfr1*/*tfr2*), for response to hypoxia (*hif1a*), iron storage (*fth*) and iron export (*fpn*). Values are presented as mean ± SD (n = 4). Differences were considered significant between control and treated groups as a, p<0.05; b, p<0.01; c, p<0.001; and between treated groups as d, p<0.05; e, p<0.01; f, p<0.001.

In the liver, we evaluated the expression of genes involved in hepcidin regulation, iron mobilization, hypoxia sensing and hematopoiesis/erythropoiesis-related factors. Overall, the severe anemia elicited a much more intense response in gene expression changes (Fig 3). Several genes associated with the iron-sensing pathways of hepcidin regulation were found to be down-regulated in the severe anemia group, such as *bmp6* and its receptor *bmpr2* and co-receptor *hjv*, and also the signaling molecules *smad4* and *smad1/5/8*. The only gene found to be upregulated was *tmprss6*, at day 4. In the light anemia group, the only changes observed were a decrease in *smad4* expression at day 4. When looking at genes involved in iron mobilization, we observed a decrease in *fth* expression in the severe anemia group. Significant up-regulations were observed for the iron transporter *tf*, as well as its receptors, *tfr1* and *tfr2*, and also of the iron exporter *fpn*, for both anemia levels. Finally, when looking at hematopoiesis-related factors, there was a significant increase in *epo* expression in both groups, although at different time points. A significant increase was also observed for *hif1a* at day 4 for both anemias, whereas for *gata2* a significant decrease was observed at days 7 and 14 in the severe anemia group and day 14 in the light anemia group.

#### Gene expression in the spleen

**Fig 4:**
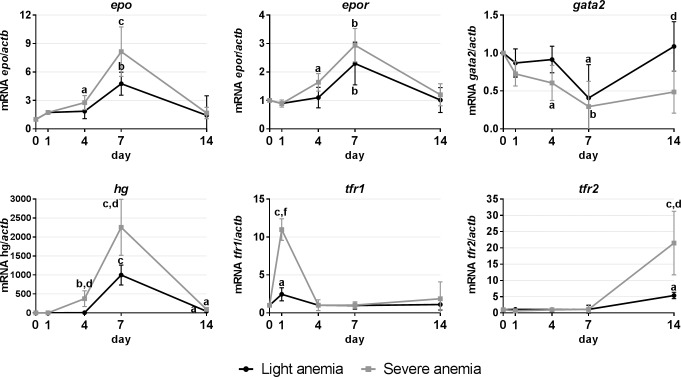
Gene expression in the spleen, after 1, 4, 7 and 14 days of experimental anemias. Genes analyzed include genes encoding hematopoietic transcription factors (*gata2*) and erythropoiesis-related factors (*epo*/*epor*/*hg*), and involved in iron homeostasis (*tfr1* and *tfr2*). Values are presented as mean ± SD (n = 4). Differences were considered significant between control and treated groups as a, p<0.05; b, p<0.01; c, p<0.001; and between treated groups as d, p<0.05; e, p<0.01; f, p<0.001.

Expression studies in the spleen were focused on genes involved in hematopoiesis (Fig 4). Similar responses were observed in response to both experimental anemias, although overall more pronounced in the severe anemia group. Expression increases were observed at days 4 and 7 for *epo*, *epor* and *hgb*, at day 1 for *tfr1* and day 14 for *tfr2*. *Gata2* was the only gene found to be down-regulated, reaching minimal levels at day 7 and with a gradual recovery towards day 14.

#### Gene expression in the head kidney

**Fig 5:**
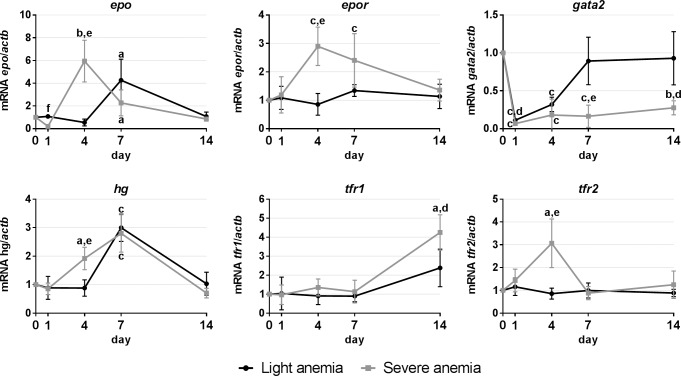
Gene expression in the head kidney, after 1, 4, 7 and 14 days of experimental anemias. Genes analyzed include genes encoding hematopoietic transcription factors (*gata2*) and erythropoiesis-related factors (*epo*/*epor*/*hg*), and involved in iron homeostasis (*tfr1* and *tfr2*). Values are presented as mean ± SD (n = 4). Differences were considered significant between control and treated groups as a, p<0.05; b, p<0.01; c, p<0.001; and between treated groups as d, p<0.05; e, p<0.01; f, p<0.001.

Expression studies in the head kidney were also focused on genes involved in hematopoiesis (Fig 5). Expression levels of *epo* were found to be increased at days 4 and 7 in the severe anemia group, and day 7 in the light anemia group. However, variations in the expression of its receptor (*epor*) were only observed in the severe anemia group, presenting increased expression also at days 4 and 7. *Gata2* levels were found to be similarly decreased at days 1 and 4 in both groups, but while levels recovered to normal values in the light anemia group at day 7, they were kept down-regulated in the severe anemia group until the end of the experiment. Variations in the expression of transferrin receptors were only observed for the severe anemia group, with an up-regulation of *tfr1* at day 14 and *tfr2* at day 4.

#### Gene expression in the posterior intestine

**Fig 6:**
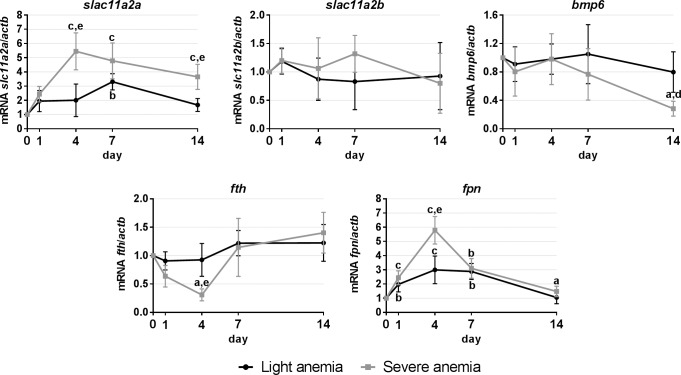
Gene expression in the intestine, after 1, 4, 7 and 14 days of experimental anemias. Genes analyzed include genes encoding transmembrane divalent metal transporters (*slc11a2a*/*b*), a possible intestine-secreted hepcidin regulator (*bmp6*), for iron storage (*fth*) and iron export (*fpn*). Values are presented as mean ± SD (n = 4). Differences were considered significant between control and treated groups as a, p<0.05; b, p<0.01; c, p<0.001; and between treated groups as d, p<0.05; e, p<0.01; f, p<0.001.

In the posterior intestine (Fig 6), expression levels of genes commonly associated with iron uptake (*slc11a2a*/*b*), export (*fpn*) and storage (*fth*) in the enterocytes were measured, as well as of a possible intestine-secreted regulator of liver hepcidin (*bmp6*). A significant increase of *slc11a2a* expression was observed in the severe anemia group, starting at day 4 and kept up-regulated until day 14. An increase was also observed at day 7 in the light anemia group. No significant changes where observed for *slc11a2b* in either group. In the severe anemia group, a down-regulation of *bmp6* and *fth* was also observed, at days 14 and 7, respectively. A similar pattern of *fpn* expression was observed for both groups, with an up-regulation starting at day 1, reaching the peak at day 4 and gradually dropping to control levels towards day 14.

### Hematological parameters and liver iron after peptide administration

**Fig 7:**
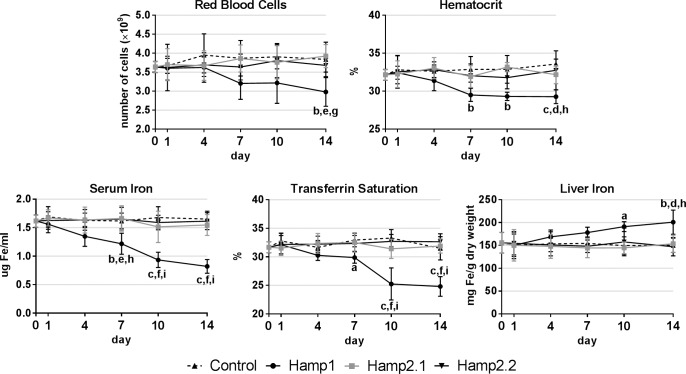
Hematological parameters and liver iron content after peptide administration. Sea bass Hamp1 and two different Hamp2 mature peptides were administered to healthy fish and hematological parameters and liver iron content were evaluated 1, 4, 7, 10 and 14 days after administration. Results are presented as mean ± SD (n = 4). Differences were considered significant between control and treated groups as a, p<0.05; b, p<0.01; c, p<0.001; between Hamp1 and Hamp2.1 as d, p<0.05; e, p<0.01; f, p<0.001; and between Hamp1 and Hamp2.2 as g, p<0.05; h, p<0.01; i, p<0.001. No significant differences were observed between both Hamp2 groups.

Hematological parameters and liver iron content were measured to evaluate the effects of administration of sea bass Hamp1 and Hamp2 mature peptides (Fig 7). Administration of Hamp1 led to steady declines of all measured hematological parameters, including red blood cell numbers, hematocrit, serum iron and transferrin saturation. On the other hand, liver iron content steadily increased during the course of the experiment. Administration of either Hamp2 failed to produce any significant effects on the hematological parameters or liver iron content, with all parameters kept similar to control levels.

## Discussion

Anemia is a very common disorder, which is characterized by abnormally low numbers of erythrocytes or hemoglobin in the blood. Iron deficiency is one of the most frequent causes for anemia, since iron is an essential element for the synthesis of hemoglobin, a crucial molecule present in the red blood cells. The causes, development and effects of iron deficiency anemia continue to be the focus of much attention in humans and other mammalian models, but have deserved little attention in other vertebrates, particularly economically important species such as teleost fish.

To better understand the molecular mechanisms of response to anemia in teleost fish, we focused our attention on the study of several genes that in mammals are known to be implicated in iron homeostasis, erythropoiesis, hematopoiesis and response to hypoxia. For that, we created two experimental models of anemia, where we induced either “light” or “severe” anemia conditions, through bleeding. To validate our experimental models, we monitored several hematological parameters as well as iron content in the liver, being the major organ of iron storage. As expected, a decrease in all measured hematological parameters was observed, clearly more accentuated in the severe anemia group. Furthermore, the severe anemia also induced a significant decrease in liver iron content, indicating an urgent need of iron mobilization to replenish the lost red blood cells. With our models thus validated, we proceeded to analyze the expression of several genes, in relevant organs known to be involved in iron homeostatis and hematopoiesis in fish, such as the liver, spleen, head kidney and posterior intestine.

One of the central players in all this is hepcidin (hamp), a small cysteine rich antimicrobial peptide considered to be the key regulator of systemic iron homeostasis [11, 12]. Unlike other vertebrates, where a single hepcidin gene seems to be the norm (with the exception of the mouse), many teleost fish, including sea bass, appear to have two hepcidin homologues, *hamp1* and *hamp2* [22, 26]. *Hamp1* has been found as a single copy gene in all studied fish species and is orthologous to the hepcidin mammalian gene, whereas *hamp2* seems to be exclusive to acanthopterygians, often presenting multiple copies. Although not always clear, several studies suggest that *hamp1* has a role focused on iron metabolism regulation, whereas the various *hamp2* have a mostly antimicrobial role [20–24, 27]. Looking into *hamp1*, we observed a decrease in expression in all tested tissues, for both anemia levels. The decreased expression in the liver is in line with our previous observations for sea bass [28], as well as with what is observed in channel catfish [29] and also mammalian models [30]. Information on the effects of anemia on hepcidin expression in the spleen, head kidney and intestine is rather scarce. However, it has been shown that these organs respond to variations in iron levels, more specifically, by increasing hepcidin expression in response to iron overload [21, 24, 31]. As such, it is logical to think that they will respond in a mirror fashion to iron deficiency, by decreasing hepcidin expression, which is what we observe in sea bass. Overall, these decreases in *hamp1* expression present a higher relevance in the liver, as the major organ of not only hepcidin constitutive expression, but also of several other genes involved in its regulation. As with mammals, it is likely that spleen, head kidney and intestine produced hamp1 has a low impact on a systemic level and is only involved in the control of local iron fluxes.

Expression of the other sea bass homolog, *hamp2*, is also decreased in the liver, and as such we cannot discard some sort of involvement in iron homeostasis, when conditions are dire (such as during severe anemia). However, *hamp2* expression increases in both head kidney and intestine. Considering the mostly antimicrobial role of hamp2 [22], this increase remains puzzling because: 1) there is no inflammatory stimuli; 2) there are no variations in the expression of genes of the inflammatory pathway (IL6/JAK/STAT pathway–data not shown); and 3) a possible crosstalk with the iron sensing pathways seems to be out of the question, as they are both down-regulated, which would lead to a decrease in hepcidin expression (as for *hamp1*). One possible explanation could be the influence of other pathways of regulation not analyzed in this study, either known ones or teleost fish specific, which certainly deserves further investigation. Another possibility is that a preventive increase in *hamp2* levels may be beneficial, since a prolonged state of anemia is bound to introduce a potential debilitative state in fish, making them more susceptible to pathogens.

In the liver, decreases in hepcidin expression were similarly accompanied by changes in the expression of genes of the regulatory pathways commonly associated with iron status, namely the HJV/BMP/SMAD and TfR mediated pathways [14]. The bone morphogenetic protein 6 (BMP6) has been recently identified as a key endogenous regulator of hepcidin and iron metabolism in mammals [32, 33]. Together with membrane-bound hemojuvelin (mHJV), it binds to the BMP receptor in the hepatocytes, leading to an up-regulation of hepcidin. However, the exact cellular source of BMP6 is still surrounded by controversy, with a group of authors claiming that the predominant source for BMP6 are the epithelial cells of the small intestine [34], whereas other authors propose that there is no role for the intestine and the liver is the key site of BMP6 production [35]. In sea bass, the liver would be not only be one of the major sites of bmp6 production, as evidenced by its observed decrease, but also of bmp6-mediated regulation, since there were significant decreases in the expression of its receptor (*bmpr2*) and co-receptor (*hjv*), as well as an increase in *tmprss6* expression, a known cleaver of membrane-bound hemojuvelin [36]. Looking downstream in this regulatory pathway, decreases in the expression of the signaling molecules *smad4* and *smad1/5/8* further reinforce the idea of an attenuation of this whole regulatory pathway.

When taking a closer look to the transferrin receptor mediated pathway, the immediate concern would be the apparent lack of *hfe* in teleost fish, which could compromise the functioning of this pathway. However, we have previously shown that despite the lack of *hfe*, the TfR-mediated pathway still responds not only to changes in the iron status, but also to inflammatory stimuli [28]. In these experiments, we observed increases in the expression of both transferrin receptors, in accordance with what is observed for mammals during iron deficiency anemia, where synthesis of the receptor is upregulated, leading to higher circulating levels of a soluble form [37, 38]. Soluble TfR (sTfR), together with serum ferritin levels, reflects different stages of iron deficiency. In the early onset of iron deficiency, there is a decline in serum ferritin, but when iron stores start to become depleted, sTfR levels begin to rise, reflecting tissue iron deficiency and iron-deficient erythropoiesis.

In line with the decrease in liver iron levels, a downregulation in the expression of the iron storage molecule *fth* and upregulation in the iron transporter *tf* and iron exporter *fpn* further suggest a decrease in storage and an increase in release and mobilization towards the organs involved in hematopoiesis.

When analyzing genes involved in erythropoiesis, we observed a very significant increase in erythropoietin (*epo*) expression. We present two possible interpretations for this data. On one hand, liver produced erythropoietin might be exerting an inhibitory effect on liver hepcidin expression. It has been proposed that erythropoietin can directly repress hepcidin in hepatocytes through EPOR-mediated regulation of the transcription factor C/EBPα [39], or indirectly through the suppression of SMAD4 and STAT3 signaling [40]. Another possibility is that this liver produced erythropoietin might be contributing to stimulate an increase in hemoglobin production in the hematopoietic organs, such as the spleen or the head kidney. Expression of the erythropoiesis factor *hif1a* seems to be in line with the increased *epo* expression. Oxygen deprivation is often associated with anemia, which in turn leads to an increased expression of oxygen sensitive transcriptional factors, such as *hif1a*. It can then bind to the hypoxia response elements present in the *epo* and *hamp* genes, enhancing or repressing their expressions, respectively [41]. Conversely, expression of the hematopoiesis transcription factor *gata2*, which is known to negatively regulate erythropoietin [42, 43], was found to be decreased.

We also looked into gene expression in the hematopoietic organs, the spleen and the head kidney. In mammals, erythropoiesis occurs in the bone marrow and is mainly regulated by the hormone erythropoietin (EPO). In teleost fish, on the other hand, the head kidney and the spleen are considered to be the major erythropoietic organs, although each organ seems to present a different importance in different fish species [37]. For sea bass, there is some evidence of the head kidney’s involvement in erythropoiesis and thrombopoiesis [44], but information on the possible involvement of the spleen is scarce. Our gene expression studies point towards the involvement of both spleen and head kidney in erythrocyte production, with the spleen seemingly assuming a more preponderant role. Similar increases in *epo* levels are observed in both organs, as well as of its receptor, *epor*, but constitutive levels of *epo* in the spleen are higher than the head kidney (14:1) and as such, the increased expression in the spleen is overall much higher. Conversely, expression levels of *gata2* are decreased, which likely results in a reduced repression of *epo*. But what really gives strength to the idea that the spleen may have a more important role in erythropoiesis in sea bass are the changes in hemoglobin and transferrin receptors expression. Although constitutive levels of *hg* are higher in the head kidney (~11:1), the biggest increase in expression occurs in the spleen, becoming millions of times higher than in the head kidney. This is accompanied by an earlier increase of *tfr1* in the spleen, against a later increase observed in the head kidney, and vice-versa for *tfr2*. *TfR1* is ubiquitously expressed, with greater relevance in erythroid cells, being deeply involved in the control of cellular iron uptake, and is essential for the development of erythrocytes [31]. On the other hand, TfR2 is mainly expressed in the liver and to a much lesser extent in erythroid cells, with a smaller contribution to cellular iron uptake and a not yet clearly defined role in the regulation of iron homeostasis [45]. Furthermore, although it is expressed at the mRNA level in erythroid cells, it does not seem to be present as a membrane protein during any phase of erythrocyte maturation [23]. As such, and at least in anemia conditions, the higher *hg* and earlier *tfr1* expressions in the spleen may indicate that it is the first erythropoietic organ to respond by increasing erythrocyte production, whereas the head kidney would later take a more unassuming role in maintaining normal erythrocyte levels.

Gene expression in the intestine, the major organ of iron absorption in vertebrates, was also investigated. Unlike mammals, where the absorption occurs mainly in the duodenal region [46], in fish absorption seems to occur mainly in the posterior regions of the intestine [47–49], hence the focus on this region. The increased expressions of *slc11a2a*, the most likely candidate for non-heme iron uptake in the posterior intestine of sea bass [49], and of the iron exporter *fpn*, together with the decrease in *fth*, suggest an increase in dietary iron absorption and release from storage from the intestinal enterocytes, either to be forwarded to the head kidney and spleen for erythropoiesis, or to the liver to replenish the iron stores. Interestingly, in conditions of severe anemia, we also observed a decrease of *bmp6* expression. Taking into account the constitutive levels of *bmp6*, highest in the liver but also considerably high in the posterior intestine, we cannot discard the possibility that, at least in teleost fish, although the liver seems to be the major regulator of *bmp6* levels, there might be some contribution by the intestinal cells when the anemia is more severe, further fueling the controversy regarding the cellular origin of BMP6 [34, 35].

Finally, we investigated the effects of sea bass synthetic peptides on the hematological parameters and liver iron content. A single Hamp1 mature peptide has been described for sea bass, whereas several Hamp2 mature peptides are expected to exist [22]. As such, we tested the effects of both Hamp1 and two different Hamp2 mature peptides. Only the administration of Hamp1 was found to have any effect on the evaluated parameters, leading to significant decreases in red blood cell numbers, hematocrit, circulating serum iron and transferrin saturation, indicating an early onset of anemia. This suggests that Hamp1 may be causing iron retention in the liver hepatocytes [16], which could be corroborated by the increased levels of iron in the liver, and most likely also in the reticuloendothelial macrophages in the hematopoietic organs [15], leading to an impaired red blood cell production, and in the intestinal enterocytes [15], limiting iron release and absorption, by blocking its target, the sole known iron exporter ferroportin [50]. This is consistent with previous observations showing that in sea bass, Hamp1 seems to have a major role in the regulation of iron homeostasis, whereas Hamp2 is almost exclusively involved in the antimicrobial response [22]. Additional experiments will be required to further clarify the exact physiological role that Hamp1 may exert on the organism.

## Conclusions

The mechanisms of response to anemia seem to be well conserved in teleost fish, namely sea bass, when compared with mammalian models, with different degrees of anemia eliciting different responses. Our results demonstrate that in response to experimental anemia, the liver assumes the main role on systemic iron metabolism regulation, through the key iron metabolism regulator hepcidin, whereas the spleen and head kidney increase their erythropoietic activity, and the intestine enhances iron absorption, presumably to provide the organism with the necessary iron for erythropoiesis. Our study indicates that each hepcidin type plays a diverse role in response to anemia, with different transcriptional responses and impact on hematological parameters and liver iron content. *Hamp1* seems to assume a role as the major regulator of iron homeostasis when enhanced erythropoiesis is required, with a possible secondary role for *hamp2*, when confronted with a severe anemia status. Further studies will be required to clarify the full extent of these roles.
